# Botulinum Toxin in Management of Limb Tremor

**DOI:** 10.3390/toxins9110365

**Published:** 2017-11-10

**Authors:** Elina Zakin, David Simpson

**Affiliations:** Department of Neuromuscular Medicine, Icahn School of Medicine at Mount Sinai, New York City, NY 10029, USA; david.simpson@mssm.edu

**Keywords:** botulinum toxin, limb tremors, muscle selection

## Abstract

Essential tremor is characterized by persistent, usually bilateral and symmetric, postural or kinetic activation of agonist and antagonist muscles involving either the distal or proximal upper extremity. Quality of life is often affected and one’s ability to perform daily tasks becomes impaired. Oral therapies, including propranolol and primidone, can be effective in the management of essential tremor, although adverse effects can limit their use and about 50% of individuals lack response to oral pharmacotherapy. Locally administered botulinum toxin injection has become increasingly useful in the management of essential tremor. Targeting of select muscles with botulinum toxin is an area of active research, and muscle selection has important implications for toxin dosing and functional outcomes. The use of anatomical landmarks with palpation, EMG guidance, electrical stimulation, and ultrasound has been studied as a technique for muscle localization in toxin injection. Earlier studies implemented a standard protocol for the injection of (predominantly) wrist flexors and extensors using palpation and EMG guidance. Targeting of muscles by selection of specific activators of tremor (tailored to each patient) using kinematic analysis might allow for improvement in efficacy, including functional outcomes. It is this individualized muscle selection and toxin dosing (requiring injection within various sites of a single muscle) that has allowed for success in the management of tremors.

## 1. Introduction

Essential tremor (ET) affects approximately 4–6% of individuals over the age of 65 [1]. Patients present with tremor characterized by persistent, bilateral, usually symmetric, postural, or kinetic tremor involving muscles of either the distal or proximal upper extremity. ET may also affect other body regions and functions including the voice, neck, lower limbs, and trunk. Over time, the severity of the tremor may worsen, and typically will affect daily tasks, including dressing, self-care, and feeding. Patients often present for evaluation and treatment once the tremor has begun to affect their quality of life. Oral therapies, including primidone, an anticonvulsant, and propranolol, a beta-adrenergic receptor antagonist, are effective in the treatment of essential tremor [2]. However, these agents reduce tremor amplitude by no more than 50%, and present a significant adverse side effect profile including dizziness, generalized fatigue, and bradycardia [3]. Additionally, about 30% of patients receive no therapeutic benefit from oral medications, leaving a relatively large population of individuals with pronounced ET untreated. Surgical options exist, including thalamotomy or implantation of either unilateral or bilateral thalamic deep brain stimulators, with Level C recommendation as ‘possibly effective’, but can only be performed in patients under the age of 75 [4]. Both pharmacologic and surgical options for the management of essential tremor, though effective for some, have their own potential for adverse events.

Local administration of intramuscular injections of botulinum toxin (BoNT) reduces excessive involuntary muscle activity and has emerged as an effective treatment for many movement disorders that are associated with muscle overactivity, including limb tremors. This article evaluates the current knowledge and evidence for the administration of botulinum toxin for limb tremors, including the process of muscle selection. This review will focus on techniques for botulinum toxin administration in limb tremors, as well as the safety/efficacy.

## 2. Review of the Literature

A tremor, which is an oscillatory movement produced by alternating or synchronous contractions of antagonistic muscles, is the most common movement disorder. Pharmacotherapy is usually not sufficient to control high-amplitude tremors, which can impair activities of daily living. Postural tremors respond more robustly to BoNT than do either kinetic or action tremors. In 1991, Jankovic, et al. reported the results of an open trial of BoNT in the treatment of 51 individuals with dystonic tremor, essential tremor, Parkinsonian tremor, peripherally induced tremor, and midbrain tremor [5]. They performed local injection for both head and limb tremor, noting that 67% of patients improved with an average latency from injection to response of 6.8 days. The average maximum duration of improvement was 10.5 weeks. This pilot study launched further investigation into the application of BoNT for selected movement disorders.

Shortly after this publication, Trosch and Pullman conducted an open-label study to determine the utility of BoNT injection in the management of severe hand tremors. They focused on forearm and arm tremors in 26 patients (12 with Parkinson disease, 14 with essential tremor), and used two clinical rating scales, subjective evaluations of function improvement and global disability, measures of weakness, and computer-assisted quantitative assessments of tremor to evaluate the effect of toxin injection after six weeks [6]. Although no change in clinical scores was noted, patients’ disability scores (as measured by the Webster Tremor and Global Disability Scales) showed statistically significant differences between pre- and post-injection. Additionally, although amplitude differences were minimal, patients reported moderate to marked subjective improvement in functional benefit after injection. Thus, the study concluded that, while no major changes in clinical ratings or objective measures were noted, BoNT injections may subjectively improve tremor in some patients, particularly those with essential tremor.

In 1996, Jankovic, et al. performed the first randomized, double-blind, placebo-controlled study to evaluate BoNT-A in essential hand tremor. They studied 25 patients who were randomized to 50 units of botulinum type A compared to placebo. They evaluated rest, postural, and kinetic tremor at two- to four-week intervals over a 16-week period, using various tremor severity rating scales, accelerometry, and subjective improvement and disability scores [7]. They noted a significant improvement on the tremor severity scale at four weeks in the toxin group as compared to placebo control, with prolonged maintenance of the effect of toxin without significant effects on functional rating scales. Tremor evaluation using postural accelerometry showed a >30% reduction in amplitude in nine of 12 toxin-treated patients.

In 2000, Pacchetti et al. reported an open-label study of BoNT in 20 patients with disabling ET who did not respond to pharmacologic therapy, using activity of daily living self-questionnaires and tremor severity scales to establish patients’ functional disability and tremor severity [8]. They noted a significant reduction in both severity and functional rating scales scores, as well as tremor amplitude reduction as measured with accelerometry and electromyography (EMG). They concluded that BoNT is safe and effective in reducing disability due to essential tremor.

In 2001, Brin et al. performed a multicenter, double-blind, placebo-controlled trial of botulinum toxin type A in essential hand tremor, studying 133 patients with ET, who were randomized to receive 50 or 100 units of botulinum toxin type A into both the wrist flexors and extensors, followed by a four-month follow-up [9]. The study showed significant improvement in postural tremor, with only minimal improvements in kinetic tremor and functional assessments.

## 3. Process of Muscle Selection in Toxin Administration for Limb Tremors

Early studies relied heavily on the clinical and electrophysiologic evaluation of patients’ tremors to localize the muscles involved. In Jankovic’s trial in 1991, careful clinical evaluation of the extremity was performed as it was held against gravity (to evaluate for postural tremor), during goal-directed movements (to evaluate kinetic tremor), or while performing specific activities such as writing (task-specific tremor). Surface electrode recording was performed on the limb in question, and muscle selection involved both agonist and antagonist groups (i.e., wrist extensors in addition to wrist flexors). Toxin was distributed into four to six different sites that were anatomically related to the muscles involved in the production of the tremor [5].

Trosch et al., working in 1994, also relied on clinical examination and EMG for tremor analysis. They used surface EMG of the flexor and extensor carpi radialis and ulnaris (FCU, FCR, ECR, and ECU), pronator teres, supinator, brachioradialis, flexor digitorum superficialis (FDS), extensor digitorum communis (EDC), biceps, and triceps muscles for each patient. Muscles that discharged rhythmically on needle EMG were injected, with almost every patient receiving wrist flexor and extensor injection [6]. Jankovic et al. used the clinical examination coupled with direct current amplification of EMG for the determination of accelerometry output for muscle selection in their randomized, double-blind, placebo-controlled study to evaluate toxin use in essential hand tremor [7]. Brin, et al. randomized patients to receive either 50 or 100 units, and used EMG guidance for BoNT administration [9]. Brin et al. and Jankovic et al. employed a fixed BoNT dosing schedule and a predetermined set of muscles. This is often currently not the case in practice, as dosages and selection of muscles injected are generally chosen individually based on tremor pattern. Pacchetti et al., in 2002, performed accelerometry and surface EMG to identify muscles with tremorogenic activity during impaired positions. They did not use EMG guidance for targeting muscles [8].

In 1997, O’Brien noted the importance of being proficient in electromyographic guidance and electrical stimulation for the improvement of the efficacy of therapy with BoNT injections [10]. More precise targeting of desired muscles for injection can be performed using these techniques, and assists the injector in preventing undesired adverse events. In using EMG guidance, the electromyographer’s objective is to record motor unit potentials that are in close proximity to the needle tip, and, in so doing, to confirm the placement of toxin in the target tissue. Crisp, full-sized, bi- or triphasic motor unit potentials with fast rise times indicate that placement is near a contracting fascicle. This technique, coupled with clinical examination, was employed in early studies evaluating the efficacy of BoNT in the management of limb tremors.

## 4. Techniques for Botulinum Toxin Administration for Limb Tremors

More recent work on the use of botulinum toxin formulations has allowed for a different set of techniques in clinical decision-making and muscle selection with regards to toxin injection. Rahimi et al., in 2015, evaluated the use of sensor-based biomechanical patterns in assisting with incobotulinumtoxinA injection in upper extremity tremors of patients with Parkinson’s disease [11]. They performed a 38-week open-label study on 528 patients with upper extremity tremor associated with Parkinson’s disease, using kinematic technology to guide muscle selection in the injection of incobotulinumtoxinA. Specific kinematic measures of tremor amplitude allowed for detailed segmentation of tremor (with sensors placed at the fingers, hand, wrist, elbow, and shoulder) into components based on the direction at each joint in the upper extremity. Using specific MatLab^®^ software, a detailed analysis of the directional contribution of each segment of the extremity was generated. Focusing on individual joints, a movement disorders neurologist used clinical judgment to determine the botulinum toxin dosage based on the kinematic measurement of tremor amplitude and the directional contribution of the tremor at each joint. This methodology tailored toxin injection to patient-specific tremor components. For the initial treatment, all participants were injected in the flexor carpi ulnaris and extensor carpi ulnaris muscles. At week 16, the Unified Parkinson’s Disease Rating Scale (UPDRS) and Fahn–Tolosa–Marin (FTM) scale (measuring tremor severity) scores showed improvement. The authors proposed that kinematics is a simple method for standardizing the assessment and treatment of upper extremity tremors in Parkinson’s disease, which allows for optimal BoNT injection and more personalized tremor therapy for this select patient population.

In 2016 Samotus et al. used kinematically determined biomechanical patterns in a study of incobotulinumtoxinA injection of 24 patients with essential tremor, who were injected every four months [1]. Motor tracking devices were placed over the forearm, wrist, elbow, and shoulder joints, which captured tremor severity in angular root mean square (RMS) amplitudes and degrees of freedom. Dosage of toxin was determined based on the injector’s interpretation of tremor severity and distribution of tremor, using kinematic recordings. Additional refinement of the technique was performed by comparing the change in tremor as measured kinematically between pre-injection to six weeks post- (peak effect of botulinum toxin) and 16 weeks post-treatment. Again, as in prior studies, the most frequently injected muscles were the FCR and ECR. This study was the first to use whole-limb kinematics to segment complex movements at multiple joints that were implicated in the tremor production. This helped target toxin injection more effectively and objectively, with targeted injections for each patient based on the unique tremor kinematics. Additional use of this kinematic technology to refine injection site selection and toxin dosage for subsequent injections was helpful for the improvement of essential tremor in these patients. The assessment of tremor movements in multiple degrees of freedom at the upper extremity joints was also useful in targeting toxin as it allowed for selection of the tremulous muscles, as opposed to only injecting the flexor and extensor wrist muscles with use of surface EMG electrodes, or a combination of surface EMG electrodes and electrical stimulation. This study concluded that the use of individualized injection parameters resulted in an improvement of tremor and disability in patients with essential tremor.

Jog and the Essential Tremor Study Team performed a prospective, randomized, double-blind, placebo-controlled multicenter study of 30 patients with essential tremor who underwent incobotulinumtoxinA versus placebo injection using TremorTrek^®^ kinematic technology to evaluate tremor amplitude (and thus judge severity) and localize muscles of involvement. They concluded that kinematic analysis-based incobotulinumtoxinA therapy decreased tremor severity and improved hand function as compared to a placebo, with marked improvement in tolerability and reduced incidence of muscle weakness [12].

Because botulinum toxin works directly at the neuromuscular junction, it is important to obtain accurate placement reaching the end plate zone (usually located at the midpoint of the muscle fiber), which helps to increase clinical response by as much as 50% [13]. The dose of botulinum toxin is usually divided between several injection sites, depending on muscle size, with reports of up to eight injection sites per muscle. Use of anatomical localization via palpation and EMG guidance has been described, though additional techniques specific to the evaluation of tremor have been employed, as described above. Precision of muscle selection with EMG and electrical stimulation has allowed for a reduced amount of toxin to be used to produce the same benefit, which has also allowed for reduced frequency of neutralizing antibody formation. Incorporation of neuromuscular ultrasound to further target muscles of interest has allowed for even more precise localization in BoNT injection. While most experienced injectors believe that careful muscle targeting improves outcomes, the American Academy of Neurology (AAN) evidence-based guidelines published in 2008 concluded that there was insufficient Class 1 evidence to recommend any muscle targeting technique for limb injections of botulinum toxin [14]. The above guidelines were put forth using an extensive literature review with two class II studies evaluating botulinum toxin administration in upper extremity essential tremor. The class of evidence for the use of botulinum toxin for the treatment of tremor is rated as level C by the AAN. Controlled comparative trials are underway to further evaluate this issue.

## 5. Safety/Efficacy of Toxin Use in Limb Tremors

The side effects of BoNT are related in part to undesired diffusion of the drug from the muscle of interest to nearby muscles/structures. This can result in inadvertent weakness of muscles that were not targeted by the clinician. Patients should be educated on the risk of possible excess weakness, usually noted within the first few weeks after toxin injection.

Contraindications to treatment with BoNT include pregnancy, known impairment of neuromuscular transmission, and myopathy, as well as the presence of prior pareses [15]. The most common adverse effects are mainly injection-site- and dose-dependent. Systemic effects are infrequent and occur after administration of 10 times the dose typically used in practice. Additionally, the formation of neutralizing antibodies is possible; studies have reported the incidence to be 0.5% to 5% of patients treated with botulinum toxin (depending on toxin subtype) [16,17].

Factors associated with the formation of neutralizing Ab resistance include total BoNT dose administered and frequency of injection. This led to the accepted dogma and FDA labeling requiring a 12-week interval between injection sessions. Notably, most of these studies of resistance were conducted with an earlier formulation of ona-BoNT containing five times the amount of protein in current formulations. More recent data show a far lower incidence of neutralizing Ab formation. This issue has increasing importance given the potential advantage of the use of “booster injections” at shorter intervals, especially in limb tremor, where careful titration of low doses may be optimal. These issues are being evaluated in controlled prospective trials.

In many of the aforementioned trials, there was a high incidence of dose-dependent adverse events, including finger weakness, pain at injection sites, hematoma formation, and paresthesias. In many of the older studies, patients self-reported a high incidence of excessive weakness, which made true rater blinding difficult to achieve. This has improved with more focused muscle targeting using kinematics (as reported in the more recent literature). Additionally, focusing toxin injection on the flexor compartment (and avoiding injection of the extensor carpi muscles, unless they were the ones causing excessive disability) allowed for preservation of finger strength in more patients, further improving patient functional outcomes in performing daily activities such as writing, using a spoon, holding a cup, and pouring liquids.

In summary, BoNT injection should be considered for patients with limb ET who have not achieved relief with oral pharmacotherapy, or in whom the drug side effect profile is poorly tolerated. After careful evaluation of the tremor, experienced clinicians, using a specific injection pattern and muscle targeting techniques such as EMG, electrical stimulation, and ultrasound, can target the toxin to the muscles of interest. These targeting techniques (along with careful anatomic palpation) allow for more effective toxin delivery, and thus minimize the possibility of toxin diffusion and neutralizing antibody formation. Novel techniques, such as quantification of tremor components and amplitude using kinematic techniques, show promise in providing even more precise localization, improved efficacy, and reduced unwanted weakness. Though no controlled data exist, one can speculate that the combination of kinematic studies, muscle visualization, ultrasonography, and confirmation with electrical stimulation will allow for an even more targeted injection approach.
